# M2I-1 disrupts the in vivo interaction between CDC20 and MAD2 and increases the sensitivities of cancer cell lines to anti-mitotic drugs via MCL-1s

**DOI:** 10.1186/s13008-019-0049-5

**Published:** 2019-06-15

**Authors:** Jianquan Li, Nanmao Dang, Nuria Martinez-Lopez, Paul A. Jowsey, Dong Huang, Robert N. Lightowlers, Fei Gao, Jun-Yong Huang

**Affiliations:** 1 Institute for Cell and Molecular Biosciences, Newcastle University, Framlington Place, Newcastle upon Tyne, NE2 4HH UK; 2 Medical Toxicology Centre, Institute of Cellular Medicine, NIHR Health Protection Research Unit, Newcastle University, Claremont Place, Newcastle upon Tyne, NE1 4AA UK; 3 Present Address: Intensive Care Unit, Guizhou Provincial People’s Hospital, Guiyang, People’s Republic of China; 4 Present Address: Department of Pediatric Critical Care Medicine, Guizhou Provincial People’s Hospital, Guiyang, People’s Republic of China

**Keywords:** M2I-1, Cyclin B1, MCL-1, Nocodazole, Taxol, Apoptosis

## Abstract

**Background:**

Drugs such as taxanes, epothilones, and vinca alkaloids are widely used in the treatment of breast, ovarian, and lung cancers but come with major side effects such as neuropathy and loss of neutrophils and as single agents have a lack of efficacy. M2I-1 (MAD2 inhibitor-1) has been shown to disrupt the CDC20-MAD2 interaction, and consequently, the assembly of the mitotic checkpoint complex (MCC).

**Results:**

We report here that M2I-1 can significantly increase the sensitivity of several cancer cell lines to anti-mitotic drugs, with cell death occurring after a prolonged mitotic arrest. In the presence of nocodazole or taxol combined with M2I-1 cell death is triggered by the premature degradation of Cyclin B1, the perturbation of the microtubule network, and an increase in the level of the pro-apoptotic protein MCL-1s combined with a marginal increase in the level of NOXA. The elevated level of MCL-1s and the marginally increased NOXA antagonized the increased level of MCL-1, a pro-survival protein of the Bcl-2 family.

**Conclusion:**

Our results provide some important molecular mechanisms for understanding the relationship between the mitotic checkpoint and programmed cell death and demonstrate that M2I-1 exhibits antitumor activity in the presence of current anti-mitotic drugs such as taxol and nocodazole and has the potential to be developed as an anticancer agent.

## Background

The spindle assembly or mitotic checkpoint (SAC) monitors the microtubule and kinetochore attachments to prevent a premature metaphase to anaphase transition and therefore helps to maintain genomic stability [1–4]. The SAC can sense any unattached kinetochores and responds by producing diffusible signals which inhibit the activity of the anaphase-promoting complex or cyclosome (APC/C) [3]. The APC/C acts as an E3 ligase and is essential for ubiquitin-mediated degradation of target proteins [1, 4–6]. Cyclin B1 is an important substrate of the APC/C and plays a critical role in facilitating mitotic entry and exit by regulating Cdk1 kinase activity [7, 8]. The MCC (Mitotic checkpoint complex) is believed to be one of the most prominent SAC inhibitory signals and consists of four components, CDC20, MAD2, BUBR1, and BUB3 [5, 9, 10]. It is assembled from the two sub-complexes, CDC20-MAD2 and BUBR1-BUB3 [10, 11]. The evidence suggests that as well as forming during prometaphase and metaphase in response to unattached kinetochores, the CDC20-MAD2 sub-complex can also be formed in prophase before nuclear envelope breakdown [6, 12, 13]. This prophase associated and SAC independent CDC20-MAD2 complex prevents the premature degradation of Cyclin B1 before the cell enters mitosis [6, 13].

Cells with depleted or down-regulated Cyclin B1 often undergo apoptosis [14, 15]. Many of the SAC components are essential, and abnormal up- or down-regulation of its activity often results in cells experiencing a prolonged mitotic arrest or prematurely exiting mitosis [3, 16]. This in turn might lead to tumorigenesis, but most of the cells will commit suicide by entering programmed cell death [17–19]. One consequence of this mitotic vulnerability has been the development of anti-mitotic drugs for cancer therapy [20–23]. In fact, traditional anti-mitotic drugs, such as taxanes, epothilones, and vinca alkaloids are widely used and have been proven to be successful in the clinical treatment of breast, ovarian, and lung cancers [24, 25]. However, because they target microtubules they lack specificity, which results in side effects such as peripheral neuropathy and rapid development of resistance, and as single agents they show a lack of efficacy [23, 26].

Blocking mitotic exit is an efficient way of inducing tumor cell death, so in principle, compounds that directly target the APC/C or MCC to cause cells to arrest in mitosis should be more efficacious and have no or fewer side effects. M2I-1 (MAD2 inhibitor-1) is the first small molecule that has been identified as an inhibitor of the CDC20-MAD2 interaction, which is an essential process in the assembly of the MCC and the inhibition of the APC/C [6, 27]. Here we report the discovery that M2I-1 enhances the sensitivity of cancer cell lines to anti-mitotic drugs which might be of use in antitumor therapy.

## Results

### M2I-1 promotes the sensitivity of cancer cell lines to anti-mitotic drugs

**Fig. 1:**
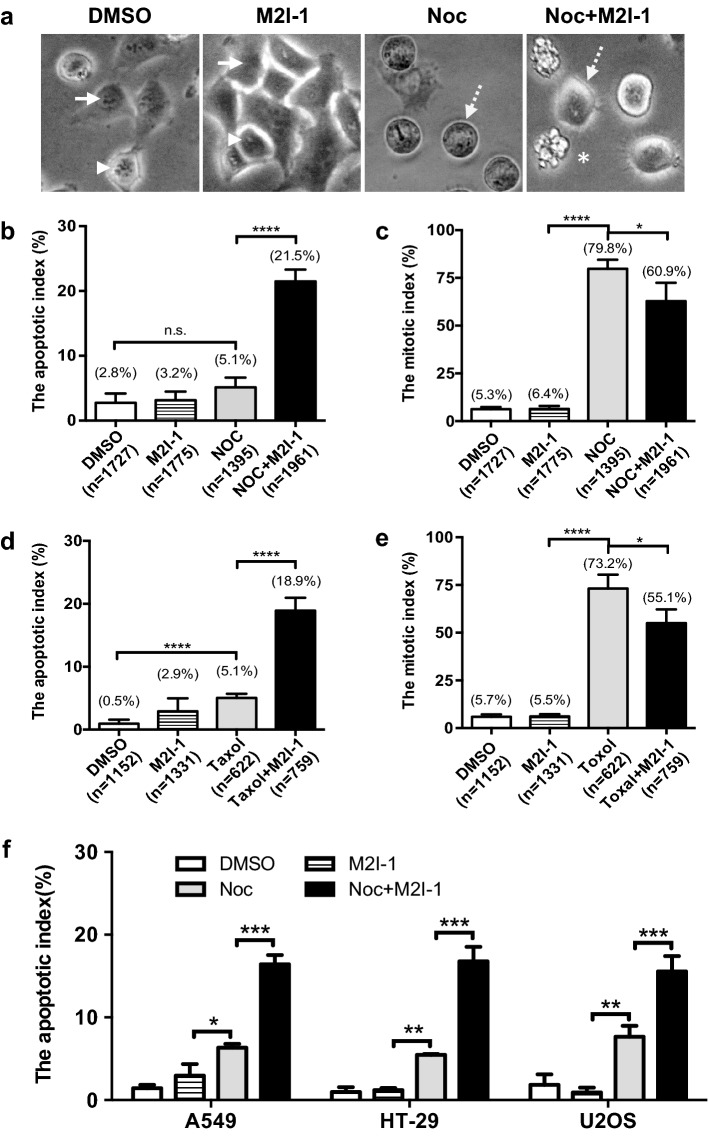
M2I-1 promotes the sensitivity of cancer cell lines to anti-mitotic drugs. HeLa cells were treated with one of 0.5% DMSO, 50 μM M2I-1, 60 ng/ml nocodazole, or 60 ng/ml nocodazole + 50 μM M2I-1 in a 24-well plate for 16 h. Digital images of cells from three or four random areas of each well were taken using a digital camera mounted on a tissue culture microscope with a 20x objective lens and used to quantify the mitotic and apoptotic indices. **a** Sample images showing non-mitotic cells (arrows), normal mitotic cells (arrowheads), mitotically arrested cells (dash line arrows), and apoptotic cells (asterisk). **b**, **d** Apoptotic indices quantified from HeLa cells treated with 60 ng/ml (equivalent to 200 nM) nocodazole or 30 nM Taxol under different circumstances as indicated. **c**, **e** Mitotic indices quantified from HeLa cells treated with 60 ng/ml nocodazole or 30 nM Taxol under the circumstances indicated. **f** Apoptotic indices quantified from A549, HT-29 and U2OS cells under the same conditions as described above. All the quantitative data were produced from four independent experiments. n, total cell numbers used for quantification; Noc, nocodazole; M2I-1, MAD2 inhibitor-1. P value: *< 0.017; **< 0.001; ***< 0.0003; ****< 0.0001

In HeLa cells a normal cell cycle lasts about 20 h with mitosis taking about 1 h [28], and the daughter cells then rapidly entering the next cell cycle. Nocodazole (NOC) is a microtubule poison, which causes depolymerisation of the microtubules and activation of the SAC to arrest the cell at prometaphase [29] and HeLa cells can tolerate treatment with 40–400 ng/ml nocodazole for up to 24 h with no significant DNA damage or cell death [30, 31], though, a prolonged mitotic arrest (> 24 h) can cause apoptosis [17, 29]. However, we have noticed that when HeLa cells were treated for 16 h with 60 ng/ml nocodazole combined with 50 μM M2I-1 (hereafter ‘NOC + M2I-1’), there was a significant (16.4%) increase in cell death compared to cells treated with 60 ng/ml nocodazole or 50 μM M2I-1 alone (Fig. 1b). The dead cells were identified by their fragmented cell body, which in many cases looks like a typical apoptotic-body (Fig. 1a, asterisk) [32, 33].

There is no significant difference in the number of cell deaths seen in cells treated with 0.5% DMSO (control), and either 50 μM M2I-1 or 60 ng/ml nocodazole alone (Fig. 1b). There is no significant difference in the mitotic index between the DMSO control and M2I-1 treated cells and as expected, about 80% of cells were arrested in mitosis in response to nocodazole treatment (Fig. 1c). In contrast, however, cells treated with NOC + M2I-1 showed a significant (18.9%) reduction in the mitotic index compared to cells treated with nocodazole alone (Fig. 1c). This increase in cell death has also been observed in cells treated with Taxol combined with M2I-1 (Fig. 1d, e). To test if this is unique to HeLa cells, various apoptotic-sensitive cell lines, HT-29, A549, U2OS were examined under the same conditions. Results showed that treatment with NOC + M2I-1 increased the rate of cell death by a varying amount in all cases (Fig. 1f). Thus it appears that M2I-1 can potentiate the sensitivity of some cancer cells to anti-mitotic drugs like nocodazole and taxol.

### The beginning of cell death occurred after prolonged mitotic arrest

**Fig. 2:**
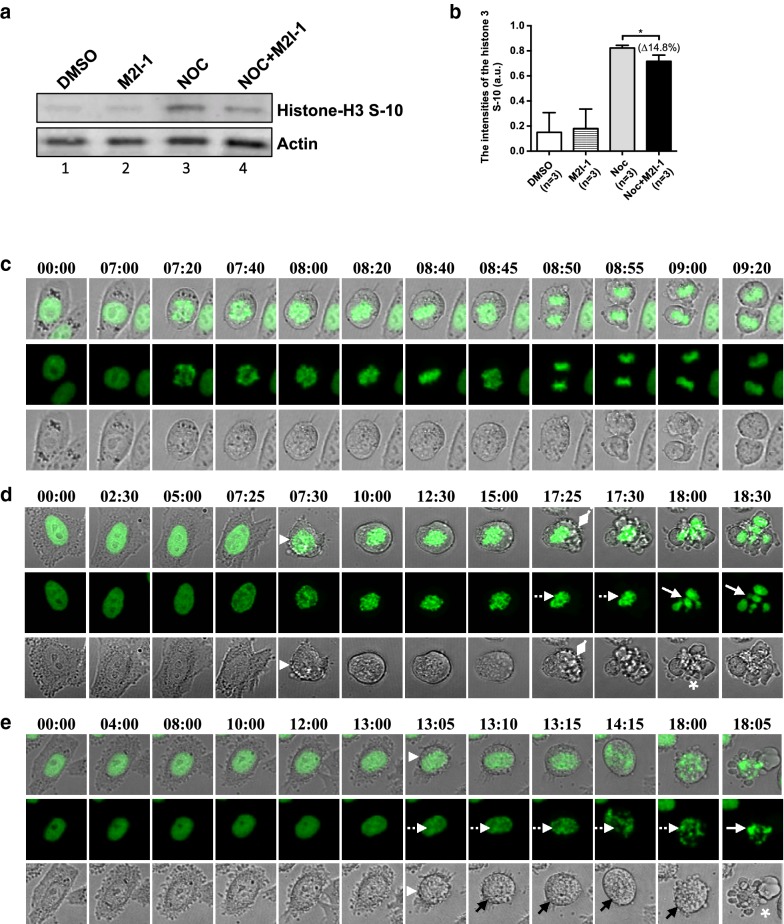
The majority of cell deaths occurred after prolonged mitotic arrest. **a** Cell extracts were prepared from HeLa cells after 16 h of the different drug treatments as described previously. The western blot membranes were probed with a rabbit polyclonal anti-phospho-histone H3 (S-10) antibody (Millipore, #06-570) (1:500 dilutions), and the actin protein bands acted as the loading control. **b** Quantitative comparison of the phospho-histone 3 S-10 bands. Results produced from three independent experiments. **c** The confocal time-lapse images showing an example of a normal/unperturbed HeLa H2B-GFP cell undergoing the cell cycle in mitosis. **d** An example of a HeLa H2B-GFP cell undergoing apoptosis after prolonged mitotic arrest by 60 ng/ml nocodazole or 60 ng/ml nocodazole + 50 μM M2I-1. The white arrowhead indicates the time point when the cytoplasmic membrane (DIC grey images) of the cell starts shrinking and rounding up with condensed chromosomes (green). The diamond heads indicate the time point that the cytoplasmic membrane began blebbing. The chromosomes displaying fragmentation and over-condensation are highlighted by white dashed arrows and normal arrows respectively. A typical apoptotic-body like morphology is highlighted with the asterisk. **e** An example of a HeLa H2B-GFP cell undergoing apoptosis at a prophase-like stage induced by treatment with 60 ng/ml nocodazole + 50 μM M2I-1. The white arrowhead highlights the time point where there is clear cell body shrinkage, dash arrows highlight the fragmenting chromosomes and normal black arrows indicate the intact cytoplasmic membrane during chromosome fragmentation. Asterisks highlight the formation of a typical apoptotic-body like morphology. The green histone 2B-GFP highlights the chromosomal morphologies and the DIC images in grey indicate the cellular boundaries and the cytoplasmic membrane. The timing of the images is indicated

Why M2I-1 in the presence of nocodazole should induce cell death is intriguing, as this contradicts what would be anticipated based on the original observations that M2I-1 disrupted the interaction between CDC20 and MAD2 and caused a weak SAC [6, 27]. We had anticipated that, at least initially, we would see less cell death when the cells were treated with NOC + M2I-1 due to the weakened SAC leading to more slippage, and this was supported by the observations that there was a reduced mitotic index, and 15% lower level of histone H3 S-10 phosphorylation in samples treated with the NOC + M2I-1 compared to samples treated with nocodazole alone (Figs. 1c, 2a, b). As the observed cell death might be caused at some stage of the next cycle after slippage, live images of a HeLa cell line over-expressing a Histone 2B-GFP fusion protein were recorded for 24 h using a Nikon A1R fully automated high-speed confocal imaging system under conditions described previously [6]. The Histone 2B-GFP signals were used to visualise the chromosomal morphology, which determines the cell cycle stage and chromosomal status; DIC images were used to reveal the morphological changes in the cytoplasmic membrane (Fig. 2c, normal cell cycle).

**Fig. 3:**
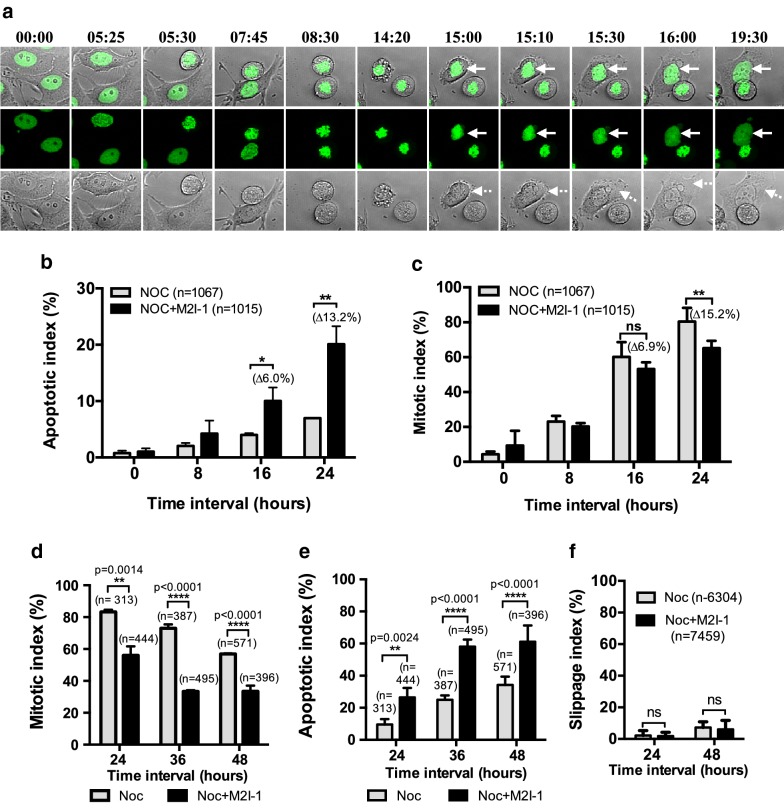
The comparison of the amount of slippage and mitotic status of the HeLa cells caused by the various drug treatments. The cells used are the same cells as for Fig. 2. **a** An example of confocal time-lapse images showing a HeLa H2B-GFP cell undergoing slippage. The white arrows indicate the chromosomes that are undergoing premature decondensation without cytokinesis having taken place. The white dash line arrows highlight the re-attachment of the cytoplasmic membrane. **b** The quantitative cell death that occurred after prolonged mitotic arrest from cells treated with 60 ng/ml nocodazole alone (grey border) or 60 ng/ml nocodazole + 50 μM M2I-1 treatment (black border). The results were produced from three independent experiments. *P value: < 0.012, **P value < 0.002. **c** Quantitative results showing the mitotic indices due to the different drug treatments (color-coded as above) at different time intervals. **P value < 0.002. **d** Quantitative results showing the mitotic indices due to the different drug treatments (black/grey borders the same as above) at different time intervals as indicated. P values shown as indicated. **e** Quantitative results showing the apoptotic indices due to the different drug treatments (color-coded the same as above) at different time intervals as indicated. P values shown as indicated. **f** Quantitative results comparing the slippage rates between different drug treatments (color-coded same as above). The data from three independent experiments were used for the quantifications. n, cell numbers used for quantification; ns, not significant

Cells were seen to round up with condensed chromosomes as they arrested at prometaphase-like states in response to the nocodazole treatment (Fig. 2d white arrowheads). The beginning of cell death was defined as a cell starting to show blebbing of its cytoplasmic membrane (Fig. 2d, diamond heads) or to display shrinkage of the cell body (Fig. 2e, black arrowheads). This was followed by the production of overcondensed chromosomes (Fig. 2d, e, white dash line arrows) or breaking of the chromosomes (Fig. 2d, e, white solid arrows), and the formation of a typical late stage apoptotic body (Fig. 2d, e, white asterisks) [34]. The slippage of a cell was determined by the decondensation of the prometaphase-like arrested chromosomes with no cytoplasmic division (Fig. 3a, arrows) and the regaining of their cytoplasmic membrane attachment (Fig. 3a, dash line arrows) [29].

The quantification of the mitotic and cell death indices using this histone 2B-GFP line (Fig. 3b, c) shows similar patterns to the previous observations (Fig. 1). There were significant increases in the cell death after 16 and 24 h of treatment with NOC + M2I-1 compared to nocodazole alone (Fig. 3b). In a separate experiment, we examined the mitotic index and apoptotic index at 24, 36 and 48 h (Fig. 3d, e). As expected the number of cells arrested in mitosis gradually reduced after 24 h (Fig. 3d), and this was correlated with the increased cell death (Fig. 3e), however, the amount of cell death induced by the presence of NOC + M2I-1 is significantly higher at each time point compared to that induced by nocodazole alone (Fig. 3e).

Given that M2I-1 induces the premature degradation of Cyclin B1 in the presence of nocodazole [6], then if the Cyclin B1 levels continue to decrease after prolonged mitotic arrest, more cells might undergo mitotic exit and escape mitotic cell death. Surprisingly though, slippage could rarely be identified in cells treated either with nocodazole alone or NOC + M2I-1 within the entire 24 h recording time, and whilst there was a slight increase in slippage events after 48 h of treatment, there were no significant differences observed between these two treatments (Fig. 3f). Instead, cell death was induced within the same cell cycle, and the majority of these cells begin to die after 16 h of mitotic arrest (Fig. 2d). Therefore, the reduced levels of histone H3 S-10 phosphorylation in samples treated with the NOC + M2I-1 compared to samples treated with nocodazole alone was a consequence of the reduced mitotic cell population caused by cell death (Figs. 1c, 2a, b). A small proportion (~ 5%) of these dead cells entered the cell death programme with a prophase-like morphology (Fig. 2e). These cells developed severe chromosomal degeneration or fragmentation first (Fig. 2e, dash line arrows) and displayed less blebbing of the cytoplasmic membrane (Fig. 2e black arrows). The typical apoptotic body-like morphology was only formed at a very late stage in the process (Fig. 2e white asterisk).

### M2I-1 induced caspase-3 cleavage in the presence of nocodazole

**Fig. 4:**
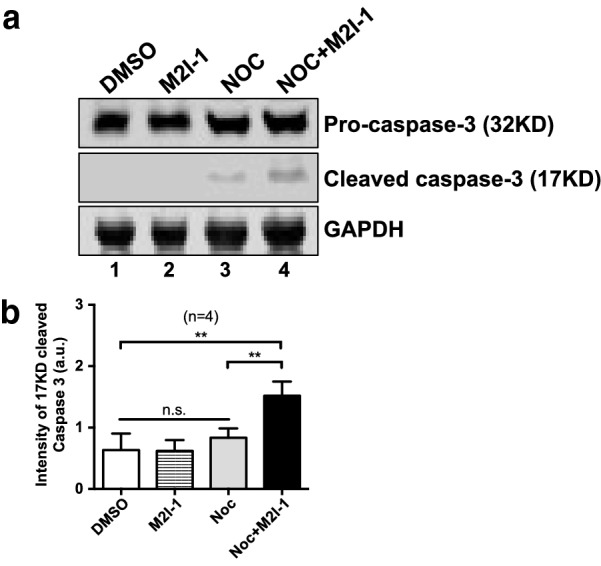
M2I-1 induced caspase-3 activity in the presence of nocodazole. **a** Cell extracts were prepared from HeLa cells under different drug treatments as indicated. 60 µg/lane of total protein from each sample was loaded for protein separation using a 10% precast SDS-PAGE tris-bis protein gel, and after western blotting the membranes were probed with an anti-caspase-3 antibody (Abcam, ab32351, 1:1000 dilutions), an anti-GAPDH antibody (Thermo Fisher Scientific, MA5-15738, 1:1000 dilutions) which acted as the loading control. Lane 1: HeLa cells treated with 0.5%DMSO (v/v) as control; lane 2: Cells treated with 50 µM M2I-1; lane 3: Cells treated with 60 ng/ml nocodazole; and lane 4: Cells treated with 60 ng/ml nocodazole + 50 µM M2I-1 for 24 h. **b** Quantitative results comparing the cleaved form of the caspase-3 from western blots of three independent experiments. P value ** < 0.0026

As the majority of the cells that died after a prolonged arrest showed typical apoptotic morphology, we examined caspase-3 cleavage to confirm that the apoptotic pathway had contributed to their cell death. The results showed that the cleavage of caspase-3 remained very low or undetectable in both the control samples treated with 0.5% DMSO and the samples treated with 50 μM M2I-1 alone (Fig. 4a, lanes 1 and 2). Samples prepared from cells treated with 60 ng/ml of nocodazole alone showed some cleavage of caspase-3 (Fig. 4a, lane 3 and b), and the level from cells treated with NOC + M2I-1 was significantly higher (Fig. 4a, lane 4 and b). Thus, the cell death induced by M2I-1 in the presence of nocodazole is associated with the caspase-3 dependent apoptotic pathway.

### The DNA damage checkpoint is less likely to contribute to this specific cell death

**Fig. 5:**
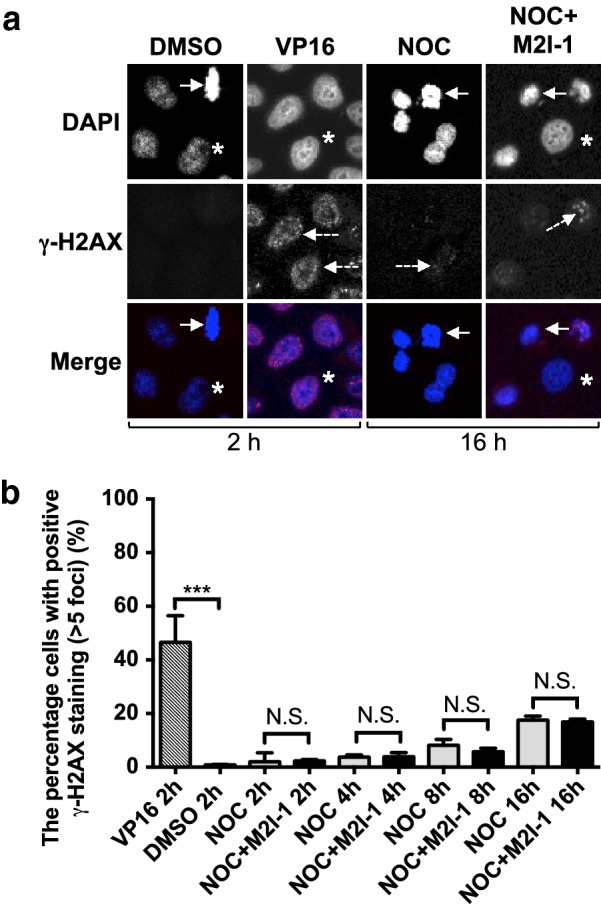
The comparison of the γ-H2AX foci formation profiles under the different drug treatment conditions. **a** Representative confocal images showing the γ-H2AX foci formed in HeLa cells at different cell cycle stages. Arrows indicate a metaphase cell treated with 0.5% DMSO for 2 h (negative control), or a prometaphase-like cell from those treated with 60 ng/ml nocodazole (NOC) alone, and 60 ng/ml NOC + 50 μM M2I-1 for 16 h respectively. Cells treated with 10 mM VP16 for 2 h act as a positive control. Asterisks highlight those un-arrested cells after NOC and NOC + M2I-1 treatment or ones from the negative control at a similar cell cycle stage to the VP16 treated cells as justified by their DNA morphologies. These cells were fixed with 4% formamide-PBS solution and stained with a primary anti-γ-H2AX antibody (Abcam, ab-2893, 1:500 dilutions). The specific fluorescent signals of the γ-H2AX foci were illustrated by a Cy5 secondary antibody (Abcam, ab6564, 1:500 dilutions) in red and were indicated by dash line arrows. DNA was stained with DAPI and is shown in blue. **b** The quantitative results comparing the DNA damage levels indicated by the percentage of cells having more than 5 γ-H2AX foci from the cell population being treated at different time intervals as indicated. Under a 40x oil objective lens, the cells from four or five random areas of the confocal images were taken to give the quantitative results. Three independent experiments were conducted to produce the data. P value *** < 0.003. NS, not significant

To examine if these cell deaths could be caused by DNA damage, we analysed the formation of γ-H2AX foci (a newly phosphorylated histone 2AX, H2A histone family member X), a typical indicator of DNA double-strand breaks [35] and used VP16 (etoposide) as a positive control as it is known to induce DNA damage [36]. The percentage of cells containing more than five γ-H2AX foci from un-arrested cells at the cell cycle stage similar to the cells treated with VP16, indicated by the morphology of their DAPI staining, has been used to represent the DNA damage level at each time point [37]. After 16 h of treatment with either NOC or NOC + M2I-1 there were insufficient numbers of un-arrested cells for quantification, so we have compared the average fluorescent intensities of the γ-H2AX foci from the mitotically arrested cells despite the fact that DNA-PKcs/CHK2 can also increase the level of γ-H2AX in mitotically arrested HeLa cells in the absence of DNA damage [37]. We anticipated that if there were additional DNA damage caused by the M2I-1 in the presence of nocodazole, we would see a higher level of γ-H2AX. The confocal images (Fig. 5a) and the quantitative results (Fig. 5b) showed that cells treated with nocodazole alone or NOC + M2I-1 have slightly more γ-H2AX foci compared to the DMSO negative control and this suggests that some DNA damage occurred under these two conditions. However, these levels of DNA damage were rather weak when compared to the levels seen in the positive control which was cells treated with VP16 for only 2 h (Fig. 5a, b). More importantly, there is no significant difference in this small amount of DNA damage at each time point examined. All of which suggests that the DNA damage pathway is not involved in the cell death induced by M2I-1 in the presence of nocodazole.

### M2I-1 in the presence of nocodazole induced the down-regulation of Cyclin B1

**Fig. 6:**
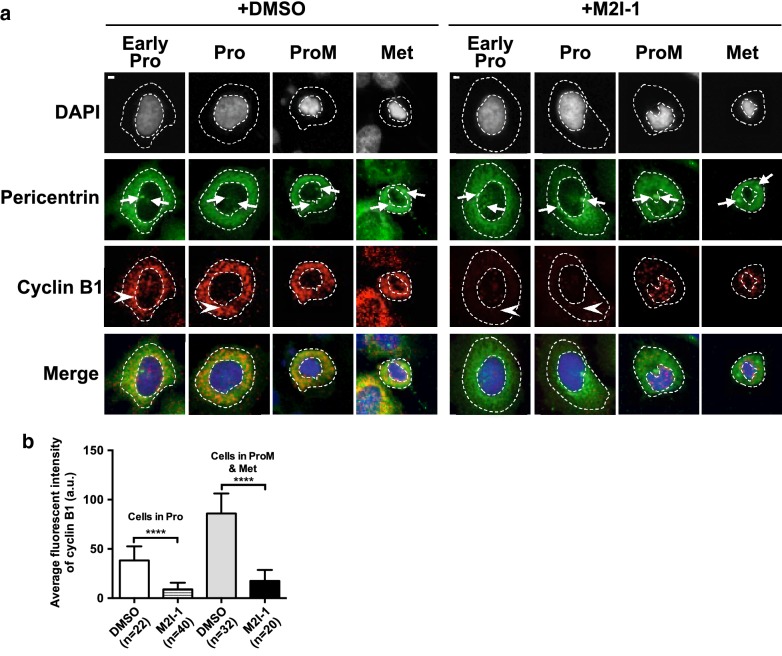
M2I-1 induced the down-regulation of Cyclin B1. **a** Representative confocal images which show the selected cell cycle stages of early prophase (Early Pro), prophase (Pro), prometaphase (ProM), and metaphase (Met) of HeLa cells after treatment with 0.5% DMSO (control) and 50 μM M2I-1 respectively. Fixed and rehydrated slides were treated with anti-Cyclin B1 antibodies for a PLA assay, after the PLA they were also probed using a pericentrin antibody and the DNA stained using DAPI. The cyclin B1 signal is in red (white arrowheads), pericentrin in green, and the DNA stained with DAPI in blue. The DNA morphologies and the centrosome status indicated by white arrows (Pericentrin staining) were used for determining the cell cycle stages as indicated. The nuclear and cytoplasmic regions are encircled by the dash lines. **b** Quantitative results comparing the levels of cyclin B1 at prophase, or prometaphase and metaphase based on single cell analysis from the cell population gathered from **a**. P value **** < 0.0001

Cyclin B1 is the regulatory subunit of Cdk1, and its level is regulated by the anaphase-promoting complex or cyclosome (APC/C) [2, 38]. Under certain circumstances Cyclin B1 has been regarded as an essential molecule in the determination of tumor cell fates [14, 34]. Previously it has been shown that targeting Cyclin B1 inhibits proliferation and sensitizes cancer cells to taxol [14, 15], and that apoptosis induced by drugs is often accompanied by the down-regulation of Cyclin B1 [14, 39]. To test if M2I-1 could prevent the accumulation of Cyclin B1, HeLa cells were treated as described before for 16 h and then fixed and antibody stained using the PLA method described previously [6] (Fig. 6a). The samples made from cells treated with M2I-1 showed significantly reduced amounts of Cyclin B1 in prophase or mitosis compared to control cells (Fig. 6a), and this was confirmed by the quantitative results (Fig. 6b). This premature Cyclin B1 degradation connected with a weakened SAC was confirmed by showing that M2I-1 disrupted the in vivo interaction between CDC20 and MAD2 in mitotic HeLa cells [6]. This was further supported by the observation of an overall reduction in Cyclin B1 detected by western blot (Fig. 8a lane 2 and 4).

### The role of “competing-networks” cannot apply to the cell death induced by M2I-1 in the presence of nocodazole

**Fig. 7:**
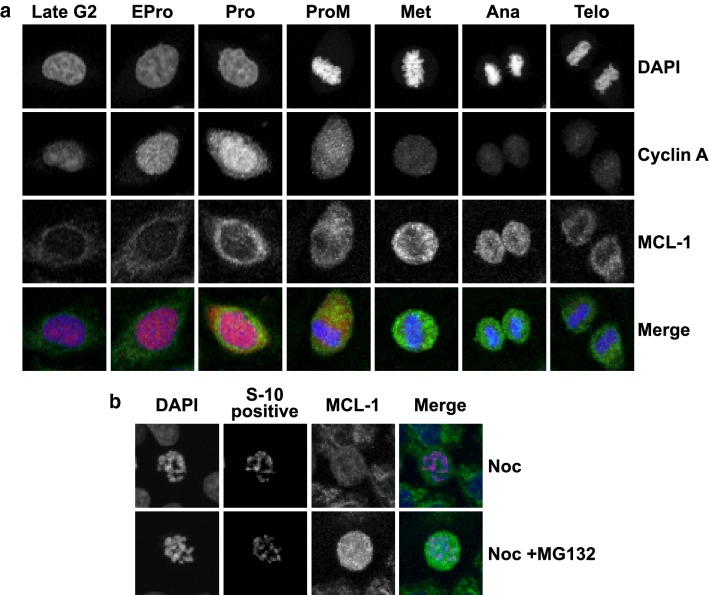
The regulation of the stabilities of MCL-1, Cyclin A in HeLa cells. **a** Representative confocal images showing the expressions and distributions of the MCL-1 (green in merged images) in unperturbed HeLa cells at different cell cycle stages as indicated. Cyclin A in red (merged images, Santa Cruz, sc-271682, mouse monoclonal anti-cyclin A (B-8) 1:500 dilutions). These cells were fixed with 4% formamide-PBS solution and stained with a primary anti-Mcl-1 (S-19, Santa Cruz, sc-819) antibody (1:500 dilutions) or anti-Cyclin A (B-8) antibody (Santa Cruz, sc-271682) (1:500 dilutions). DNA was stained with DAPI (blue in merged images). The distributions of the fluorescent signals of Cyclin A throughout the cell cycle used for timing the cell cycle stages for comparison purpose. **b** Representative confocal images showing the expressions of MCL-1 (green) in nocodazole arrested (top panel) or nocodazole and MG132 arrested (bottom panel) HeLa cells. DNA was stained with DAPI (blue in merged images) and the mitotic status highlighted by staining with the phosphor-histone H3 S-10 antibody (red, Cell signalling, 9706, 1:200 dilutions). HeLa cells were treated with nocodazole or nocodazole + MG132 for 8 h

MCL-1 (myeloid cell leukemia 1) is a pro-survival member of the Bcl-2 family of proteins [40]. It prevents the oligomerisation of the pro-apoptotic proteins Bak and Bax on the mitochondrial membrane, and so, stops the release of cytochrome C thus avoiding the activation of caspase [40, 41]. In unperturbed normal HeLa cells the expression of MCL-1 is cell cycle regulated, its levels increase during mitosis and peak around metaphase and early anaphase, but remained low in interphase (Fig. 7a). In response to the nocodazole provoked SAC it is degraded (Fig. 7b bottom panel) and that degradation can be prevented by MG132 (Fig. 7b bottom panel).

The degradation of MCL-1 is associated with the triggering of apoptosis in many different types of cells [42, 43] and its phosphorylation by Cyclin B1-dependent Cdk1 kinase facilitates this degradation [44]. It has been suggested that the accumulation of Cyclin B1 caused by an active SAC facilitates the degradation of MCL-1 to promote apoptosis, and vice versa [45]. A more recently proposed “competing-networks” model suggests that the rise of an as yet undefined death signal competes with the survival signal generated by the reduction of Cyclin B1 to determine whether the cell dies in mitosis or exits and returns to interphase [20, 34].

**Fig. 8:**
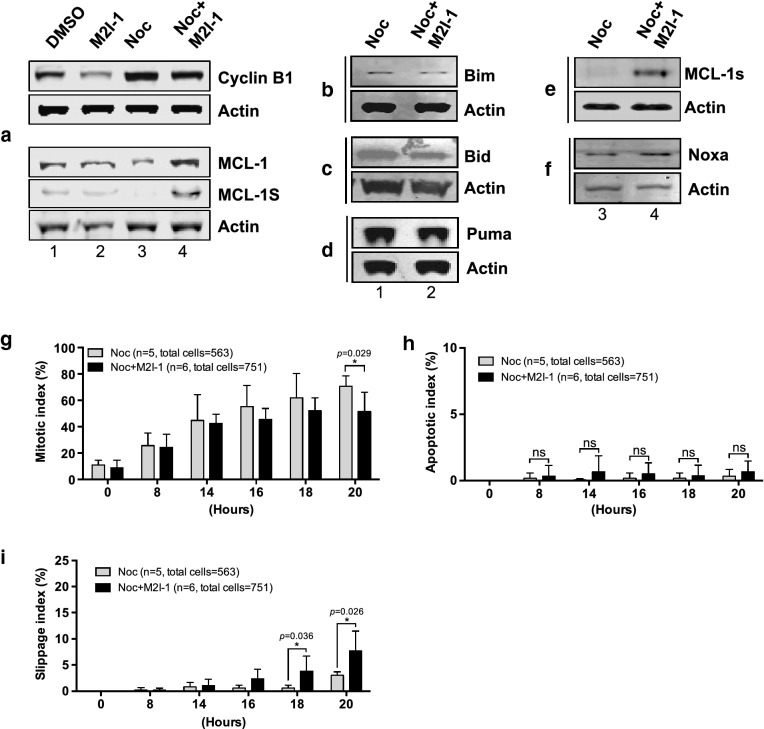
The regulation of the stability of MCL-1, cyclin B1, and other pro- or anti-apoptotic proteins in HeLa and MCF-7 cells. **a** Western blots of cell extracts prepared from HeLa cells after treatment with 0.5% DMSO, 50 μM M2I-1, 60 ng/ml nocodazole, and 60 ng/ml nocodazole + 50 μM M2I-1 respectively and probed with a rabbit polyclonal anti-cyclin B1 antibody (H-433) antibody (sc-7520); a mouse monoclonal anti-actin (AC-15) antibody (ab6276) was used as the loading control. **b**–**f** Western blot results showing the expression levels of the pro-apoptotic proteins BIM, BID, PUMA, NOXA, and MCL-1s in HeLa cells. Cell extracts were prepared from HeLa cells after treatment with nocodazole alone or nocodazole + M2I-1 as described before. Actin bands were used as the loading controls. MCF-7 cells were treated using the same conditions as described in Fig. 2e apart from the determination of the chromosomal morphologies, which was highlighted by staining with 0.2 μ M SiR-DNA. Digital images were taken after 20 h incubation for quantification of the mitotic (**g**), apoptotic (**h**), and slippage indices (**i**). n: The number of the parallel experiments and the total cell numbers used for quantification. P value * < 0.026, 0.029, and 0.036 respectively

**Fig. 9:**
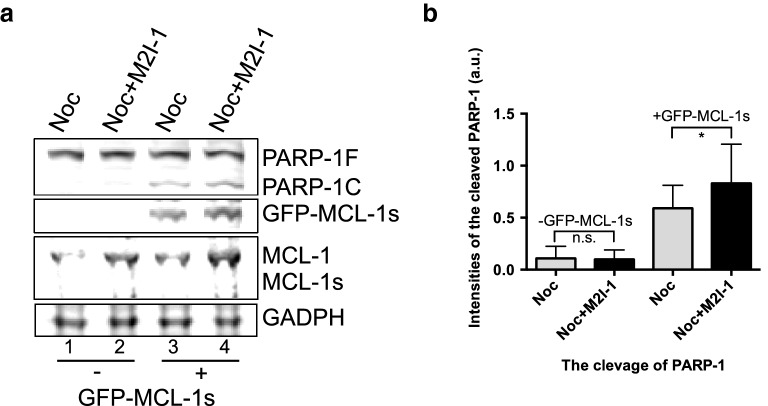
Ectopically expressed mGFP-MCL-1s sensitizes the MCF-7 cells to M2I-1 in the presence of nocodazole. **a** Western blot results showing the expression levels of PARP-1F (full length) and its cleaved fragment PARP-1C, MCL-1, MCL-1s and the exogenous GFP-MCL-1s. GAPDH was used as the loading control. **b** The quantification of the cleaved PARP-1C under the experimental condition used in **a**. Data was quantified from three independent experiments after being normalised with the loading control. Paired *t* test. P value: * < 0.014

We, therefore, examined the protein profiles of Cyclin B1 and MCL-1 from the samples described above (Fig. 8a). As expected, when Cyclin B1 was stabilized by the treatment with nocodazole alone, it was associated with a significant reduction of MCL-1 (Fig. 8a, lane 3), and the lowered Cyclin B1 induced by the combined drugs, is associated with an elevated MCL-1 (Fig. 8a, lane 4). This is a surprise when the cell death observed previously (Fig. 1b, d) is also considered. The levels of the pro-apoptotic proteins BIM, BID, and PUMA remained relatively unchanged or even slightly reduced (Fig. 8b–d), but the level of NOXA increased marginally (Fig. 8f lane 4). However, the levels of an anti-survival protein, MCL-1s (a short form variant of the full-length MCL-1 which acts to sequester the function of MCL-1), increased when the cells were treated with NOC + M2I-1 compared to nocodazole alone (Fig. 8a lane 4 and e lane 4). Therefore, the increased levels of the pro-apoptotic proteins MCL-1s and NOXA might antagonize the increased level of MCL-1. In order to test this speculation, live-cell images were recorded examining the behaviour of a cell line (MCF-7) which lacks the expression of MCL-1s [46], in response to prolonged mitotic arrest in the presence of nocodazole and M2I-1 or nocodazole alone. The experiment was conducted using the same conditions as described in Fig. 2 apart from determination of the chromosomal morphologies which was illustrated by staining with 0.2 μM SiR-DNA (Tebu-Bio, UK) [47]. As with HeLa cells, the mitotic index began to reduce in the group treated with both drugs after 16 h of incubation, and this became significant at 20 h compared to the cells treated with nocodazole alone (Fig. 8g). However, in contrast to HeLa cells, M2I-1 failed to induce cell death in the presence of nocodazole in MCF-7 cells over the time course of the observations (Fig. 8h), and the reduced mitotic index in MCF-7 cells after double drug treatment is caused by cell slippage (Fig. 8i). As MCL-1s is a short form and the consequence of an alternative splicing event of the full length MCL-1 pre-mRNA [48, 49], it is impossible to design siRNA experiments that directly and specifically target MCL-1s for depletion in HeLa cells, and so we transfected the MCF-7 cell line with the plasmid DNA of pCMV-AN-mGFP-MCL-1s so that it would express the exogenous GFP-MCL-1s protein. These transfected cells (Fig. 9, lane 3 and 4) along with the normal MCF-7 cells (Fig. 9, lane 1 and 2) were then treated with the drugs as described above, and the samples were prepared for western blot analysis. As MCF-7 cells also lack the expression of caspase-3 [50, 51], we examined the cleavage of PARP-1 (Poly [ADP-ribose] polymerase 1) to evaluate the apoptotic activity, as it is a well-established biomarker for cell death [52, 53]. The lack of expression of the endogenous MCL-1s in these samples (Fig. 9, lane 1–4), and the expression of the exogenous GFP-MCL-1s fusion protein in the transfected MCF-7 cells (Fig. 9, lane 3 and 4) were confirmed using a specific anti-MCL-1 antibody. The endogenous MCL-1 in these MCF-7 cells behaved the same as it had done in the HeLa cells. Its levels increased when cells were treated with the combined drugs NOC + M2I-1 compared to the cells treated with nocodazole alone (Fig. 9a, lanes 2 and 4 compared to lanes 1 and 3). Similarly, the level of exogenous GFP-MCL-1s is also increased in the sample treated with the combined drugs (Fig. 9a, lane 4 compared to lane 3). The results show that the level of the cleaved PARP-1 increased significantly in the sample treated with NOC + M2I-1 compared to the sample treated with nocodazole alone in the cells transfected with GFP-MCL-1s (Fig. 9a, lane 3 and 4 and b). In contrast, only very low levels of PARP-1 and no significant difference in its cleavage could be detected from the normal MCF-7 cells after treatment with the same conditions as above (Fig. 9a, lanes 1 and 2). Thus, MCL-1s is the main factor that antagonizes the elevated level of MCL-1 and causes the cell death induced by M2I-1 in the presence of nocodazole or taxol.

## Discussion

M2I-1 (MAD2 inhibitor-1) is the first small molecule that has been identified which disrupts the CDC20-MAD2 interaction both in vitro and in vivo, an essential process in the assembly of the MCC [6, 27]. We have previously reported that M2I-1 can prevent the formation of the CDC20-MAD2 complex both at prophase before NEBD (nuclear envelope break-down) and at prometaphase and metaphase [6]. We have also found that the disruption of the interaction between CDC20 and MAD2 induced by the M2I-1 treatment correlated with the premature degradation of Cyclin B1 at both stages (Fig. 6a, b) [6]. Intriguingly, we show here that M2I-1 could significantly increase the sensitivity of several lines of cancer cells to anti-mitotic drugs such as nocodazole and taxol both within 24 h or beyond (Figs. 1, 3). It has been believed that when a cell is in a prolonged mitotic arrest, a gradually declining level of Cyclin B1 and a stabilised level of MCL-1serve as a survival signal which competes with an as yet undefined death signal to determine whether the cell dies in mitosis or exits and returns to interphase [20, 34, 45]. Our results, however, suggest that in HeLa cells under the current experimental conditions, the accumulation of Cyclin B1 with a reduced MCL-1 would not trigger apoptosis; moreover, an elevated MCL-1 and lowered Cyclin B1 would not directly trigger slippage either (Figs. 1, 2, 3, 6, 8). More interestingly, M2I-1 in the presence of nocodazole or taxol could induce cell death in cells with a low level of Cyclin B1 and stabilized MCL-1 under a weakened SAC (Figs. 1, 3, 6, 8). This phenomenon cannot be explained by the “competing-networks” model [20]. Most likely, the premature degradation of Cyclin B1 caused by the M2I-1 treatment throughout the cell cycle combined with the microtubule network disruption caused by nocodazole or taxol reduced the cells fitness. The increased levels of the pro-apoptotic proteins MCL-1s and NOXA antagonized the pro-survival function of MCL-1 and triggered cells into undergoing apoptosis (Figs. 8, 9).

## Conclusion

We have shown that as a single agent M2I-1 cannot cause cancer cell death, but it can significantly increase many cancer cells sensitivity to anti-mitotic drugs, such as nocodazole and taxol within the same cell cycle. This might prove to be significant, as it would increase the clinical efficacies of current drugs such as taxanes, epothilones, and vinca alkaloids and potentially reduce the length of treatment as well as the dose used. It might also slow any developing resistances and the possibility of relapse or new tumorigenesis after chemotherapy using current anti-mitotic drugs, though this has yet to be tested. We have also discovered some important molecular mechanisms for understanding the relationships between the mitotic checkpoint and programmed cell death.

## Materials and methods

### Antibodies and reagents

*Primary antibodies used in this project are* Rabbit polyclonal anti-CDC20 antibody (Abcam, ab26483); mouse monoclonal anti-p55 CDC (E-7) (Santa Cruz Biotech, sc-13162); rabbit polyclonal anti-full length MAD2 (Convance, PRB-452C); mouse monoclonal anti-cyclin B1 (GNS) (Santa Cruz, sc-245); mouse monoclonal anti-cyclin A (B-8) (Santa Cruz, sc-271682); mouse monoclonal anti-actin antibody (Abcam, ab6276); mouse monoclonal anti-GADPH antibody (Thermo Fisher Scientific, MA5-15738); rabbit polyclonal anti-caspase-3 antibody (Abcam, ab32351); rabbit polyclonal anti-phospho-histone 3 (S-10) antibody (Millipore, #06-570); and rabbit polyclonal anti-GFP antibody [Santa Cruz, sc-8334 (GP-FL)]; rabbit polyclonal anti-γ-H2AX (S-139) antibody (Abcam, ab-2893); GFP-Trap A geta-20 (ChromoTek, 70112001A); rabbit polyclonal anti-Mcl-1 (S-19) antibody (Santa Cruz, sc-819); rabbit polyclonal anti-pericentrin 1&2 antibody (Abcam, ab4448); rabbit polyclonal anti-BID (FL-195) antibody (Santa Cruz, sc-11423); mouse monoclonal anti-BIM (H-5) antibody (Santa Cruz, sc-374358); rabbit polyclonal anti-NOXA (FL-54) antibody (Santa Cruz, sc-30209) and monoclonal anti-PUMA (G-3) antibody (Santa Cruz, sc-374223); rabbit monoclonal anti-human PARP-1 antibody (46D11) (Cell Signaling Technology).

*Secondary antibodies* goat polyclonal secondary antibody to rabbit IgG-H + L (DyLight 488) (Abcam, ab96899), goat anti-rabbit polyclonal IgG-H&L (Cy5), pre-adsorbed (Abcam, ab6564), IRDye 680 donkey anti-mouse (926–322227; LI-COR Biosciences), and IRDye 800CW donkey anti-mouse (926–32212; LI-COR Biosciences).

*Duolink reagents* Duolink In Situ PLA probe anti-Rabbit PLUS, Duolink In Situ PLA probe anti-Mouse Minus, Duolink In Situ Detection Reagents Red (the Duolink assay reagent kits are distributed by Sigma-Aldrich).

*Chemicals* M2I-1 (ChemBridge Corporation), CelLytic™ MT Cell Lysis Reagent (Sigma-Aldrich, C3228), Protease inhibitor cocktail (Sigma-Aldrich, p8340), Thymidine (Sigma-Aldrich, Cas-358801), Nocodazole (Sigma-Aldrich, Cas-31430-18-9), Taxol (Paclitaxel, T7402) (Sigma-Aldrich), VP16 (etoposide) (Sigma-Aldrich, E1383), DMEM (Sigma-Aldrich), DAPI (4′, 6-Diamidine-2′-phenylindole dihydrochloride) (Sigma-Aldrich, D9542), DMSO (Dimethylsulfoxide, Santa Cruz, sc-358801). Odyssey blocking solution (Li_Cor Biosciences UK Ltd). SiR-DNA and Verapamil (Tebu-Bio).

### Plasmid DNA and cell transfection

The original pcDNA3-flag-MCL-1s (human) was kindly provided as a gift by Professor Jeehyeon Bae’s lab at Chung-ANG university of South Korea. The plasmid DNA was used as a template for PCR (polymerase chain reaction) to generate the MCL-1s DNA fragment incorporated with Sgf1 and Mul1 restriction enzyme sites at its 5′ and 3′ ends respectively. This PCR fragment of Sgf1-MCL-1(s)-Mul1 was subsequently subcloned into a mammalian expression vector, pCMV6-AN-mGFP, at Sgf1 and Mul1 sites, in frame with and downstream of the mGFP sequence. The primer pair used for MCL-1s PCR, was 5′ forward: GAATTCGCGATCGCCATGTTTGGCCTCAAAAGAAACGC and 3′ reverse: GAATTCACGCGTTCACAGTAAGGCTATCTTATTAGATATGC.

The plasmid DNA transfections were performed using the LipofectamineTM3000 kit (ThermoFisher Scientific, UK) according to the commercial protocol. Briefly, MCF-7 cells were grown in a T75 flask with 14 ml of an antibiotic-free DMEM medium up to 70% confluence. 1 µg/ml of pCMV6-AN-mGFP-MCL-1s plasmid DNA was used for transfection. The solution containing the DNA lipid complex was added to MCF-7 cells for incubation at 37 °C supplied with 5% CO_2_ for 24 h. The transfected cells were harvested and resuspended in 12 ml of fresh complete medium and were split equally and seeded into 6 wells of a 6-well plate. The cells were used for drug treatment after all of them attached to the wells, which normally took about 6–8 h.

### Cell culture conditions and treatments

The HeLa cell line overexpressing histone 2B-GFP, A549, HT29, U2OS and the RPE1CCNB1-Venus cells (a gift from Jonathan Pines’ lab, RPE1, a human retinal pigment epithelial cell line) was cultured in DMEM medium (Dulbecco’s Modified Eagle Medium) as described before [6]. Cells were treated with 30 nM Taxol [34], 10 μM VP16, and 60 ng/ml (equivalent to 200 nM) nocodazole [5] or with 50 µM M2I-1 [27], or M2I-1 and nocodazole together in 0.5% DMSO for the lengths of time discussed in the results. Subsequently the treated or untreated HeLa cells were fixed using 1 ml cold (− 20 °C) methanol [8] and left at room temperature for 5 min, or were fixed with 1 ml of 4% formamide-PBS (phosphate-buffer saline) solution for 10 min at room temperature and then stored at − 20 °C for later use. Cell extracts prepared using the same drug treatments but with increased culture volumes were used for western blotting.

### Protein gel and western blotting

The cell extracts for gel electrophoresis, and western blotting were prepared as described before [6]. Cells were lysed in an appropriate amount of CelLytic™ MT Cell Lysis Reagent containing 1 × protease inhibitor cocktail on ice with agitation for 30 min. Samples of the cell lysates were separated using a precast 10% Bis–Tris SDS-PAGE gel (sodium dodecyl sulfate polyacrylamide gel electrophoresis), and were then transferred onto a nitrocellulose membrane for western blotting. The membranes were blocked in 1x Odyssey blocking solution for 1 h and then probed with appropriate primary antibodies in Odyssey blocking solution (1:500 dilution) at room temperature for 2 h or at 4 °C overnight with agitation. The membrane was then incubated with a secondary antibody solution of IRDye 680 donkey anti-mouse, or IRDye 800CW donkey anti-mouse, at 1:2000 dilutions for one and a half hours. The fluorescence signals were detected using a CCD scanner (Odyssey; LI-COR Biosciences) according to the manufacturer’s instructions.

### Immunofluoresence for γ-H2AX

HeLa cells on a coverslip prepared as described above were washed three times in 1× PBS, and blocked with a blocking solution of 5% BSA (bovine serum albumin) in 1xPBS for an hour at room temperature followed by incubation with a primary mouse anti-human γ-H2AX antibody at a dilution of 1:500 in blocking solution. The cells were incubated overnight at 4 °C. Cells were washed twice in 1× PBS and once with 1× PBST (1× PBS + 0.1% Tween-20), for 5 min each. This was followed by incubation with a Cy5 goat anti-mouse IgG secondary antibody at a dilution of 1:500 in blocking solution containing an appropriate amount of DAPI at room temperature for 90 min in the dark. After being washed twice in 1× PBS, and once with 1× PBST and air-dried as above the cells on the coverslips were mounted with an appropriate amount of 50% glycerol-PBS solution and sealed using nail vanish for later use.

### PLA fluorescent immunostaining

The Duolink based proximity ligation assay (PLA) was conducted as described previously [6] with the following changes. The paired primary antibodies used were a mouse monoclonal anti-Cyclin B1 and a rabbit polyclonal anti-Cyclin B1 (H-433).

### Confocal imaging and quantification of the fluorescent complexes

The stained HeLa cells were scanned using a Leica TCS SP2 laser scanning confocal system same as described before [6].

The live images of the histone 2B-GFP in HeLa cells or MCF-7 cells stained with 0.2 μM SiR-DNA to illustrate the chromosome morphologies were recorded using a Nikon A1R fully automated high-speed confocal imaging system with a time interval of 5 min over 24 h.

ImageJ, Photoshop, and NIS-elements were used for quantification, editing of the fluorescent intensities of the complex or live imaging processing where appropriate.

## Data Availability

All data generated or analyzed in this study are included in the article.

## Abbreviations

M2I-1: MAD2 inhibitor-1
MCC: mitotic checkpoint complex
APC/C: anaphase-promoting complex or cyclosome
SAC: spindle assembly or mitotic checkpoint
NOC: nocodazole
H2AX: H2A histone family member X
VP16: etoposide
DMSO: dimethylsulfoxide
MCL-1: myeloid cell leukemia 1
PARP-1: poly [ADP-ribose] polymerase 1
NEBD: nuclear envelope break-down
PLA: proximity ligation assay
PCR: polymerase chain reaction
RPE1: a human retinal pigment epithelial cell line
DMEM: Dulbecco’s modified eagle medium
SDS-PAGE: sodium dodecyl sulfate polyacrylamide gel electrophoresis
BSA: bovine serum albumin
PBS: phosphate-buffer saline
